# Biochemical and Hematological Predictors of Mortality in Thai Patients with COVID-19

**DOI:** 10.3390/medsci13040281

**Published:** 2025-11-24

**Authors:** Supaporn Wiwattanakul, Rutchaporn Taweerutchana, Kitsarawut Khuancharee, Pornparn Rojanasang, Pongwut Suwannarat, Prapaporn Panichchob, Pornsuk Romputtan, Nopparut Teravaninthorn, Nichapat Wiriyakunakorn, Monpat Chamnanphon

**Affiliations:** 1 Department of Pathology, HRH Princess Maha Chakri Sirindhorn Medical Center, Faculty of Medicine, Srinakharinwirot University, Nakhon Nayok 26120, Thailand; supapowi@g.swu.ac.th (S.W.); rojanasang@yahoo.com (P.R.); arm_msmc@yahoo.com (P.S.); p_panich14@hotmail.com (P.P.); pornsukpui@gmail.com (P.R.); nopparut.teravaninthorn@g.swu.ac.th (N.T.); nichapat.wir@g.swu.ac.th (N.W.); 2 Division of Endocrinopathy, Department of Medicine, Faculty of Medicine, Srinakharinwirot University, Nakhon Nayok 26120, Thailand; rutchaporn@g.swu.ac.th; 3 Department of Preventive and Social Medicine, Faculty of Medicine, Srinakharinwirot University, Nakhon Nayok 26120, Thailand; kitsarawut@g.swu.ac.th

**Keywords:** COVID-19, biochemical markers, hematological markers, mortality predictors, clinical biomarkers

## Abstract

Background: Coronavirus disease (COVID-19), caused by SARS-CoV-2 infection, presents a broad spectrum of clinical manifestations, ranging from asymptomatic cases to severe and fatal outcomes. Studies have shown that laboratory parameters fluctuate in patients with COVID-19, and these parameters serve as valuable biomarkers for monitoring disease progression. This study examines the relationship between changes in biochemical and hematological markers and patient survival among early COVID-19 cases. Materials and methods: In this retrospective cohort study, data from adult (≥18 years) hospitalized COVID-19 patients with positive PCR results at HRH Princess Maha Chakri Sirindhorn Medical Center, Srinakharinwirot University, Nakhon Nayok, Thailand, between March and December 2021, were analyzed. Univariate and multivariate logistic regression analyses were conducted on mortality-related laboratory parameters. All measures are reported as adjusted odds ratios (aORs) with 95% confidence intervals (CIs). Results: The cohort included 397 patients with pneumonia (median age: 52.2 years (IQR: 40.5–64.6); 61.96% female). Among them, 42 patients (10.58%) succumbed during hospitalization, with a median hospital stay of 12.92 days (IQR: 10.03–15.94). Independent mortality predictors were identified as follows: age (aOR = 1.11; 95% CI: 1.04–1.19; *p =* 0.002), potassium (aOR = 6.27; 95% CI: 1.31–29.93; *p* = 0.021), creatinine (aOR = 1.62; 95% CI: 1.05–2.50; *p =* 0.028), hemoglobin A1c (aOR = 1.96; 95% CI: 1.30–2.97; *p =* 0.001), and red cell distribution width (aOR = 1.45; 95% CI: 1.05–2.02; *p =* 0.026), respectively. Furthermore, patients with lower platelet counts had a notably higher risk of mortality (aOR = 0.98; 95% CI: 0.97–0.99; *p =* 0.001). Conclusions: Our findings suggest that age, potassium, creatinine, hemoglobin A1c, red cell distribution width, and platelet count are significant predictors of mortality risk in patients with COVID-19. Clinicians should consider these biochemical and hematological markers critically before initiating treatment for COVID-19 patients.

## 1. Introduction

In March 2020, the World Health Organization (WHO) declared COVID-19, a disease first identified in an outbreak in Wuhan, China in December 2019, a global pandemic. Coronavirus disease (COVID-19) is an infectious disease caused by the novel SARS-CoV-2 virus, a member of the coronavirus (CoV) family. COVID-19 is a positive-sense single-stranded RNA (+ssRNA) virus with a single linear RNA segment encapsulated within a membrane envelope. The disease primarily spreads through person-to-person contact via contaminated respiratory droplets from coughing or sneezing, but direct contact with saliva and sputum can also transmit it. COVID-19 symptoms present across a broad spectrum of clinical manifestations [1]. They can range from asymptomatic to very severe symptoms, including severe pneumonia or even organ failure. Common symptoms include headache, loss of smell and taste, nasal congestion, runny nose, cough, muscle pain, sore throat, fever, diarrhea, and breathing difficulties. As of January 2023, the WHO Coronavirus (COVID-19) Dashboard reports 663,001,898 cumulative cases and 6,707,959 deaths worldwide [2]. In Thailand, COVID-19 has also become a significant public health concern. The Department of Disease Control in Thailand reports 4,725,885 cumulative cases and 34,697 deaths [3]. Therefore, it is crucial to accurately assess the severity of COVID-19 to manage patients effectively and prevent fatal complications. Numerous have investigated factors associated with the severity of COVID-19. One such factor, C-reactive protein (CRP), is a nonspecific acute-phase protein produced in the liver in response to IL-6, increasing during inflammation, infection, and tissue damage. Several studies have revealed elevated CRP levels in severe and fatal COVID-19 patients [4] due to a “cytokine storm,” marked by the excessive release of various pro-inflammatory cytokines. The neutrophil-to-lymphocyte ratio (NLR) is another inflammatory marker that correlates with systemic inflammation. Elevated NLR is associated with an increase in neutrophils and lymphocyte apoptosis, which may lead to immunological anomalies in the body. Numerous studies have found a significant association between NLR and both disease severity and mortality [5,6]. SARS-CoV-2 infection and the resulting inflammation can lead to pulmonary capillary and endothelial damage, causing platelet (PLT) aggregation, microthrombus formation, and increased platelet consumption [7]. Studies have shown that platelet counts decrease in severe cases compared to those in non-severe cases [8,9].

Additionally, prothrombin time is often prolonged in severe cases compared to non-severe cases [10,11] due to decreased platelet counts. D-dimer, a product of fibrin degradation by plasmin, is a marker of thrombosis and fibrinolysis and rises in the plasma when fibrinolysis occurs. Studies have demonstrated a significant association between elevated D-dimer levels and acute respiratory distress syndrome (ARDS) development [12]. However, Lei et al. [13] found that D-dimer levels were not significantly different between patients admitted to the intensive care unit (ICU) and those in non-ICU care (*p* = 0.99). Therefore, it is essential to monitor complete blood count (CBC) parameters in COVID-19 patients upon admission. We conducted this study to analyze these markers in our hospital, as, to our knowledge, no similar research has been undertaken here previously; the primary objective was to identify biochemical and hematological parameters that predict mortality among hospitalized COVID-19 patients in Thailand, aiming to provide locally relevant clinical biomarkers to assist in early risk stratification and improve patient management.

## 2. Materials and Methods

### 2.1. Patients’ Demographics

In this retrospective cohort study, all data records of adult (≥18 years old) hospitalized COVID-19 patients with positive PCR results were evaluated at HRH Princess Maha Chakri Sirindhorn Medical Center, Srinakharinwirot University, in Nakhon Nayok, Thailand, between March 2021 and December 2021, prior to the emergence of the Omicron variant and widespread vaccination campaigns in Thailand. The Ethics Committee of the Faculty of Medicine, Srinakharinwirot University (SWUEC/E/M-013/2565E, date of approval: 17 February 2022), ethically approved this study. The patient’s informed consent form was waived because the study is a retrospective review of existing patient medical records.

### 2.2. Data Collection

This study utilized detailed medical records of patients with laboratory-confirmed COVID-19 disease hospitalized at HRH Princess Maha Chakri Sirindhorn Medical Center, Nakhon Nayok. Comprehensive data were extracted, including hematological profiles (e.g., leukocyte, lymphocyte, and platelet counts), biochemical markers (e.g., electrolytes, liver enzymes, and renal function tests), coagulation parameters (e.g., prothrombin time and D-dimer levels), and arterial blood gas analyses. The records encompassed a wide range of diagnostic and monitoring parameters crucial for assessing disease progression and patient outcomes. Data extraction focused on ensuring accuracy and completeness to facilitate robust analysis and meaningful comparisons between survivors and non-survivors. It is important to note that detailed information regarding the specific treatments administered during hospitalization, including medication types, oxygen therapy, and ICU care protocols, was not consistently documented in patient medical records and therefore was not included in this analysis.

### 2.3. Statistical Data Analysis

Descriptive statistics were employed to summarize the data, with continuous variables represented as medians accompanied by interquartile ranges (IQRs), contingent upon the distribution of the data. The analytical approach articulated categorical variables in terms of frequencies and percentages. The laboratory parameters, encompassing biochemical and hematological markers, were subjected to comparative analysis between COVID-19 survivors and non-survivors utilizing the Mann–Whitney U test and the Kruskal–Wallis test (median, interquartile range; IQR). Univariate and multivariate logistic regression analysis assessed associations between potential risk factors and non-survivors, yielding odds ratios (ORs) and 95% confidence intervals (CIs). Logistic regression was chosen due to limitations in the available time-to-event data, focusing on mortality as a binary endpoint. We acknowledge that this approach limits the ability to account for the timing of events, which represents a recognized limitation of the study. Statistical significance was *p* < 0.05. We used STATA software version 14 (StataCorp LP, College Station, TX, USA) for all analyses, ensuring robust data evaluation and analysis.

## 3. Results

### 3.1. Clinical Characteristics

This investigation categorizes critical COVID-19 patients into two groups: survivors and non-survivors. Table 1 delineates the demographic characteristics of both COVID-19 survivors and non-survivors. The distribution of gender and age exhibited statistically significant disparities between the survivor and non-survivor groups. The overall sample comprised 246 females (61.96%) and 152 males (38.04%). The median length of hospital stay was 12.88 days (IQR 10.03–15.64) in survivors versus 15.08 days (IQR 9.98–25.24) in non-survivors (*p* = 0.073). Clinical parameters, including systolic blood pressure (SBP), diastolic blood pressure (DBP), body temperature (T), and heart rate (HR) revealed no statistically significant differences between the two groups.

### 3.2. Biochemical Parameters

Table 2 highlights notable variations in key biochemical parameters when comparing survivors and non-survivors. Non-survivors exhibited lower levels of total protein, albumin, and albumin/globulin (A/G) ratio (*p* < 0.001). Liver function markers were also significantly altered, with non-survivors showing higher total bilirubin, direct bilirubin, and aspartate transaminase (AST) (*p* < 0.001). Non-survivors showed markedly elevated inflammatory markers, including lactate dehydrogenase (LDH), C-reactive protein (CRP), and procalcitonin (*p* < 0.001). Renal function was impaired in non-survivors, as evidenced by higher blood urea nitrogen (BUN) and creatinine levels (*p* < 0.001). Electrolyte imbalances were evident, with non-survivors showing lower sodium (Na^+^), chloride (Cl^−^), and bicarbonate (HCO3^−^) (*p* < 0.001), but higher potassium (K^+^) levels (*p* < 0.001) and increased anion gap (*p* < 0.001). We found no significant differences for globulin, indirect bilirubin, alanine transaminase, alkaline phosphatase, hemoglobin A1c, lactate, pH, pCO_2_, pO_2_, and SO_2_ (Supplementary Table S1).

### 3.3. Hematological Parameters

Analysis of hematological parameters revealed significant differences between survivors and non-survivors (Table 2). Non-survivors had higher D-dimer levels (*p* < 0.001), indicating an increased risk of coagulopathy. Anemia was more pronounced in non-survivors, as evidenced by lower hemoglobin level (*p* < 0.001), hematocrit (*p* = 0.0003), and red cell count (*p* = 0.002). Red cell distribution width (RDW) was higher in non-survivors (*p* < 0.001), suggesting greater red cell size variability. Platelet counts were lower in non-survivors (*p* < 0.001), while mean platelet volume (MPV) was slightly higher (*p* = 0.047). The white cell count increased in non-survivors (*p* = 0.010), displaying a higher neutrophil percentage (*p* < 0.001) and a lower lymphocyte percentage (*p* < 0.001). Prothrombin time, activated partial thromboplastin time, mean corpuscular volume (MCV), mean corpuscular hemoglobin (MCH), and mean corpuscular hemoglobin concentration (MCHC) showed no significant differences (Supplementary Table S1).

The multivariate analysis in Table 3 highlights RDW and platelet count as independent predictors of mortality in severe COVID-19. Key Predictors of Mortality: Age (aOR = 1.11, *p* = 0.002), potassium (aOR = 6.27, *p* = 0.021), creatinine (aOR = 1.62, *p* = 0.028), hemoglobin A1c (aOR = 1.96, *p* = 0.001), RDW (aOR = 1.45, *p* = 0.026), and platelet count (aOR = 0.98, *p* = 0.001) were significant independent predictors of mortality among COVID-19 patients.

## 4. Discussion

This study aims to identify laboratory parameters that differentiate COVID-19 survivors from non-survivors, offering critical insights for predicting disease severity and improving patient management. The findings align with previous research, highlighting the prognostic significance of specific biochemical and hematological markers in managing COVID-19.

SARS-CoV-2 utilizes the angiotensin-converting enzyme 2 (ACE2) receptor to enter host cells. This receptor appears in various tissues, including hepatocytes and bile duct epithelial cells [14,15,16,17]. The binding of SARS-CoV-2 to ACE2 can disrupt the renin–angiotensin–aldosterone system (RAAS), leading to an imbalance that may contribute to hyponatremia in COVID-19 patients [18,19]. Elevated potassium levels observed in non-survivors likely reflect underlying renal impairment, metabolic acidosis, and cellular damage, all of which disrupt potassium homeostasis. Hyperkalemia is recognized as a critical prognostic marker in COVID-19, as it adversely affects cardiac excitability and conduction, significantly increasing the risk of fatal arrhythmias and mortality. Close monitoring and management of potassium abnormalities are therefore essential in critically ill COVID-19 patients. Previous studies have consistently identified hypoproteinemia and hyponatremia as adverse prognostic markers in COVID-19 patients [19,20]. Hyponatremia, commonly caused by dehydration, sepsis, or kidney dysfunction, is associated with increased mortality due to fluid imbalances and neurological complications [19]. During cytokine storms, hyponatremia may result from the non-osmotic release of vasopressin driven by elevated IL-6 levels [21]. The hypoproteinemia observed in COVID-19 patients may result from increased protein degradation and inadequate nutritional intake. Severe COVID-19 induces a hypercatabolic state, leading to excessive protein loss. Additionally, symptoms such as anorexia, vomiting, and diarrhea, reported in a significant proportion of patients, reduce food intake and exacerbate protein depletion [20]. Hyperkalemia, often arising from metabolic acidosis, kidney dysfunction, and cellular breakdown, disrupts potassium homeostasis. This imbalance adversely affects cardiac excitability and conduction, significantly increasing the risk of fatal arrhythmias and death [22].

Significant differences in key biochemical parameters between COVID-19 survivors and non-survivors highlight the critical role of systemic inflammation and organ dysfunction in disease outcomes. The findings of elevated inflammatory markers such as C-reactive protein (CRP) and neutrophil-to-lymphocyte ratio (NLR) in non-survivors align with previous studies demonstrating their association with COVID-19 severity and mortality [4,5,6]. Similarly, reductions in platelet counts observed in our cohort corroborate reports linking thrombocytopenia with severe disease [7,8,9]. Elevated D-dimer levels further support the role of coagulopathy in poor outcomes as described in earlier research [11,12]. These consistencies validate the prognostic value of these biomarkers in monitoring COVID-19 progression and risk stratification. Non-survivors exhibited markedly lower levels of total protein and albumin, indicating hypoalbuminemia and impaired liver function. Hypoalbuminemia has been identified as a predictor of poor outcomes in critically ill patients, including those with COVID-19, as it reflects heightened systemic inflammation, reduced nutritional status, and hepatic dysfunction [23]. The significantly lower albumin-to-globulin ratio in non-survivors further indicates an imbalance in protein metabolism, commonly driven by chronic inflammation or hepatic impairment. Elevated levels of total and direct bilirubin in non-survivors suggest potential liver dysfunction or biliary obstruction. These abnormalities could result from direct viral injury to hepatocytes, hypoxic damage due to respiratory failure, or drug-induced liver injury associated with COVID-19 treatment regimens [24]. Such findings align with previous studies that have linked hyperbilirubinemia to severe disease outcomes [23]. Increased AST and LDH levels in non-survivors indicate tissue injury and systemic inflammation. Elevated AST often reflects hepatic damage but can also be attributed to myocardial or skeletal muscle injury [24,25]. LDH, a marker of cell damage, is associated with disease severity and multi-organ dysfunction in COVID-19 patients [23]. Elevated LDH has been consistently linked to higher mortality risk, underscoring its prognostic value [24,26]. Inflammatory markers, such as C-reactive protein (CRP) and procalcitonin, are markedly elevated in non-survivors. CRP, an acute-phase protein, is directly associated with systemic inflammation and correlates strongly with disease severity and adverse outcomes [27]. Procalcitonin (PCT), traditionally a biomarker for bacterial infections, has been observed to rise in severe COVID-19 cases. Cytokine dysregulation and potential secondary bacterial infections cause this elevation. Studies have shown that elevated PCT levels in COVID-19 patients are associated with increased disease severity and mortality [28,29,30]. These biochemical markers collectively underscore the interplay between systemic inflammation, organ dysfunction, and disease severity in non-survivors. Monitoring these parameters can provide critical insights into patient prognosis and inform tailored therapeutic strategies.

Analysis of hematological parameters has revealed significant differences between survivors and non-survivors of COVID-19, highlighting underscoring the role of immune dysregulation, systemic inflammation, and hematologic imbalances in determining disease severity and mortality. Non-survivors exhibited significantly lower Hb, HCT, RBC counts, platelet counts, and lymphocyte percentages. These findings are consistent with the anemia and thrombocytopenia observed in critically ill COVID-19 patients, which likely result from a combination of inflammation-mediated suppression of erythropoiesis, increase platelet consumption of, and direct viral effects on hematopoietic progenitors [31]. Low platelet counts in non-survivors are indicative of thrombocytopenia, which has been strongly associated with severe disease and higher mortality rates in COVID-19 [8]. Mechanisms contributing to thrombocytopenia include immune-mediated destruction of platelets, increased consumption in microthrombi, and direct damage to megakaryocytes by the virus [31,32]. Conversely, non-survivors showed significantly elevated red cell distribution width (RDW) and mean platelet volume (MPV), both of which are markers of increased hematologic stress and inflammation. Elevated RDW has been identified as a predictor of poor outcomes in COVID-19, reflecting anisocytosis and increased erythrocyte turnover caused by systemic inflammation [33,34]. Similarly, increased MPV, an indicator of activated platelets, is associated with heightened thrombotic risk in severe COVID-19 [35]. Higher white blood cell counts and neutrophil percentages in non-survivors point to a systemic inflammatory response and immune dysregulation. Neutrophilia, combined with lymphopenia, is a hallmark of severe COVID-19 and is strongly associated with cytokine storms and impaired adaptive immune responses [31]. The elevated neutrophil-to-lymphocyte ratio (NLR) in non-survivors has also been highlighted as a robust prognostic marker for mortality [35]. Thrombocytopenia observed in COVID-19 may be caused by immune complexes destroyed through molecular mimicry or by increased platelet consumption due to damage to lung tissue and pulmonary endothelial cells [36]. SARS-CoV-2 causes endothelial dysfunction and promotes thrombus formation, leading to increased platelet consumption [36]. While many studies report elevated D-dimer levels in severe cases, our study found inconsistencies in D-dimer levels across patients, potentially reflecting variability in disease course and timing of sample collection [37]. Prior research supports the use of D-dimer as a marker for thrombotic complications, particularly for individuals at risk of ARDS [38]. Markedly elevated D-dimer levels in COVID-19 non-survivors highlight a hypercoagulable state consistent with disseminated intravascular coagulation, despite no significant differences in prothrombin time or activated partial thromboplastin time [37,39]. Elevated D-dimer reflects increased fibrinolysis and thrombin generation due to endothelial injury, systemic inflammation, and cytokine storms [40]. Microvascular thrombosis, frequently observed in severe cases, contributes to multi-organ failure and poor outcomes [40]. High D-dimer levels are strongly associated with mortality and serve as a critical prognostic marker [41]. Anticoagulation therapy, especially in patients with elevated D-dimer levels, has shown improved outcomes, underscoring the importance of monitoring coagulation markers in risk stratification and management [42].

The observed differences in laboratory parameters between survivors and non-survivors highlight the potential of a multi-marker approach for risk stratification in COVID-19. Integrating biochemical, hematological, and inflammatory markers into predictive models may enhance the accuracy in assessing disease severity and mortality risk [43]. These findings suggest that liver dysfunction, renal impairment, inflammation, and coagulopathy are key factors associated with poor outcomes. In contrast, ALT, globulin, and blood gas values showed no significant differences, indicating their limited predictive value in this cohort. This finding supports the development of clinical guidelines that incorporate routine lab tests into COVID-19 management protocols.

## 5. Limitations and Future Directions

As a retrospective, single-center cohort, our study is subject to inherent limitations, including potential selection bias and incomplete data on important treatment variables, such as medication regimens, oxygen therapy, and intensive care management protocols. The lack of comprehensive treatment details limits our ability to assess their impact on patient outcomes fully and may introduce residual confounding. Potential confounding inherent to comparisons between survivors and non-survivors should also be acknowledged. Additionally, our sample size, although adequate for primary analyses, may limit statistical power for specific subgroup evaluations. The relatively small number of events (42 deaths) may further limit the statistical power and increase the risk of overfitting. Furthermore, due to data constraints, time-to-event analyses such as Kaplan–Meier survival curves and Cox proportional hazards regression could not be performed, which represents an inherent limitation of the study. Furthermore, the single-institution setting may affect the generalizability of our findings to other populations or clinical environments. The data were collected in 2021, before the Omicron variant and before vaccines were universally available; thus, outcomes and key predictors might differ in more recent populations with different viral variants or vaccination status. Another limitation of our study is that it focused primarily on laboratory values obtained at admission or during hospitalization without external validation in independent cohorts. Consequently, the predictive value of these biomarkers needs confirmation in future longitudinal, multicenter, prospective studies that incorporate larger and more diverse patient populations. Moreover, data on the timing of COVID-19 diagnosis relative to symptom onset and hospitalization were unavailable, which may influence the interpretation of temporal changes in biomarker levels. Information on circulating SARS-CoV-2 Variants of Concern (VOCs) during the study period was also not collected, potentially affecting disease severity and laboratory results. Moreover, ferritin levels—a key marker of cytokine storm and hyperinflammation—were not consistently measured across our cohort, limiting the evaluation of their prognostic significance. Moreover, no formal correction for multiple testing was applied, which may affect the interpretation of statistical significance, particularly in multivariate analyses presented in Table 3. Given the evolving nature of SARS-CoV-2 variants and clinical management, future longitudinal, multicenter prospective studies incorporating detailed treatment data, timing of diagnosis, viral genomic information, and comprehensive inflammatory markers, such as ferritin, are warranted to validate and expand upon our findings.

## 6. Conclusions

Non-survivors of COVID-19 exhibited significant derangements in biochemical and hematological parameters compared to survivors. Elevated liver enzymes (AST, bilirubin), inflammatory markers (CRP, procalcitonin, LDH), renal markers (BUN, creatinine), and D-dimer, along with lower albumin, hemoglobin, hematocrit, and platelets, were associated with mortality.

## Figures and Tables

**Table 1:** Demographic data of COVID-19 patients between survivors and non-survivors.

Variable	Survivors Median (IQR), (n = 355)	Non-Survivors Median (IQR), (n = 42)	*p*-Value
Gender			
-Female	227 (63.94%)	19 (45.24%)	0.018
-Male	128 (36.06%)	23 (54.76%)	
Age group (years)	50.50 (39.50–61.60)	69.45 (63.80–76.80)	<0.001
-18–59	253 (71.27%)	8 (19.05%)	<0.001
-≥60	102 (28.73%)	34 (80.95%)	
SBP (mmHg)	127 (116–141)	137 (118–145)	0.149
DBP (mmHg)	80 (71–89)	76 (67–90)	0.227
T (°C)	36.80 (36.50–37.40)	36.90 (36.50–37.60)	0.905
HR (bpm)	90 (80–100)	90.5 (82–100)	0.714

**Table 2:** Laboratory data of COVID-19 patients between survivors and non-survivors.

Parameter	Survivors Median (IQR), (n = 355)	Non-Survivors Median (IQR), (n = 42)	*p*-Value
Total Protein (g/dL)	7.30 (6.90–7.70)	6.95 (6.40–7.30)	<0.001
Albumin (g/dL)	4.10 (3.80–4.40)	3.50 (3.20–3.90)	<0.001
Albumin/Globulin Ratio	1.30 (1.10–1.40)	1.00 (0.90–1.30)	<0.001
Total Bilirubin (mg/dL)	0.33 (0.25–0.48)	0.51 (0.33–0.81)	<0.001
Direct Bilirubin (mg/dL)	0.21 (0.15–0.28)	0.31 (0.21–0.53)	<0.001
Aspartate Transaminase (U/L)	36.00 (27.00–54.00)	52.50 (34.00–87.00)	<0.001
Lactate Dehydrogenase (U/L)	231.50 (181.50–312.50)	311.50 (252.00–458.50)	<0.001
C-Reactive Protein (mg/L)	13.00 (5.03–41.55)	64.60 (41.90–114.00)	<0.001
Procalcitonin (ng/mL)	0.07 (0.05–0.14)	0.76 (0.02–1.73)	<0.001
Sodium (Na^+^) (mmol/L)	137.30 (134.60–139.30)	133.85 (132.10–136.90)	<0.001
Potassium (K^+^) (mmol/L)	3.60 (3.40–3.90)	3.90 (3.50–4.30)	<0.001
Cl^−^ (mmol/L)	101.00 (98.00–103.00)	97.50 (94.00–100.00)	<0.001
HCO_3_^−^ (mmol/L)	22.55 (20.60–24.20)	20.25 (18.60–22.20)	<0.001
Anion Gap	14.00 (12.00–16.00)	17.00 (13.00–21.00)	<0.001
Blood Urea Nitrogen (mg/dL)	10.20 (8.10–13.20)	17.75 (12.10–32.00)	<0.001
Creatinine (mg/dL)	0.80 (0.66–0.98)	1.21 (0.88–2.12)	<0.001
D-dimer (µg/mL)	0.46 (0.28–0.83)	1.185 (0.69–3.075)	<0.001
Hemoglobin (g/dL)	13.00 (11.80–14.30)	11.45 (8.90–13.70)	<0.001
Hematocrit (%)	39.20 (35.50–42.40)	34.40 (26.10–40.20)	<0.001
Red Cell Count (×10^6^ cells/µL)	4.80 (4.36–5.29)	4.27 (3.21–5.09)	0.002
RDW (%)	13.20 (12.60–14.20)	14.60 (13.50–17.20)	<0.001
White Cell Count (×10^3^/µL)	6.00 (4.70–7.90)	7.15 (5.60–10.80)	0.010
Neutrophil (%)	63.50 (54.40–72.40)	77.40 (68.50–84.20)	<0.001
Lymphocyte (%)	26.80 (19.50–36.60)	14.55 (9.30–23.30)	<0.001
Platelets (×10^3^/mm^3^)	231.00 (183.00–289.00)	177.50 (117.00–232.00)	<0.001
MPV (fL)	10.20 (9.60–10.80)	10.45 (9.90–11.20)	0.047

**Table 3:** Multivariate logistic regression analysis of the variable factors associated with non-survival.

Factors	Odd Ratio (95%CI)	*p*-Value
Length of hospital stay (days)	0.96 (0.92–1.01)	0.154
Age (years)	1.11 (1.04–1.19)	0.002
DBP (mmHg)	1.05 (0.99–1.11)	0.072
Na^+^ (mmol/L)	0.90 (0.80–1.02)	0.089
K^+^ (mmol/L)	6.27 (1.31–29.93)	0.021
Creatinine (mg/dL)	1.62 (1.05–2.50)	0.028
Alkaline Phosphatase (U/L)	1.00 (0.99–1.00)	0.052
Hemoglobin A1c (%)	1.96 (1.30–2.97)	0.001
RDW (%)	1.45 (1.05–2.02)	0.026
Platelets (×10^3^ cells/ mm^3^)	0.98 (0.97–0.99)	0.001

## Data Availability

The original contributions presented in this study are included in the article/supplementary material. Further inquiries can be directed to the corresponding author.

## Supplementary Materials

The following supporting information can be downloaded at: https://www.mdpi.com/article/10.3390/medsci13040281/s1, Table S1: Laboratory data of COVID-19 patients between survivors and non-survivors.
