# Supplementary material for: Biochemical and Hematological Predictors of Mortality in Thai Patients with COVID-19
Source: Med Sci (Basel). 2025 Nov 24;13(4):281. doi: 10.3390/medsci13040281 (PMC12734457; doi:10.3390/medsci13040281)
Supplement: Supplementary file 1 [file medsci-13-00281-s001.zip › medsci-3846529-supplementary.pdf]

Supplementary Table S1. Laboratory data of COVID-19 patients between survivors and non-survivors

Laboratory parameters	Total, Median (IQR), n=397	Survivors, Median (IQR), n=355	Non-survivors, Median (IQR), n=42	*P*-value
**Biochemical parameters**				
Total Protein (g/dL)	7.3 (6.9-7.6)	7.3 (6.9-7.7)	6.95 (6.4-7.3)	<0.001
Albumin (g/dL)	4.1 (3.7-4.3)	4.1 (3.8-4.4)	3.5 (3.2-3.9)	<0.001
Globulin (g/dL)	3.3 (3-3.5)	3.3 (3-3.5)	3.3 (3-3.8)	0.3157
A/G Ratio (Albumin/Globulin)	1.3 (1.1-1.4)	1.3 (1.1-1.4)	1 (0.9-1.3)	<0.001
Total Bilirubin (mg/dL)	0.34 (0.26-0.5)	0.33 (0.25-0.48)	0.51 (0.33-0.81)	<0.001
Direct Bilirubin (mg/dL)	0.21 (0.16-0.3)	0.21 (0.15-0.28)	0.31 (0.21-0.53)	<0.001
Indirect Bilirubin (mg/dL)	0.13 (0.09-0.2)	0.13 (0.09-0.2)	0.19 (0.08-0.28)	0.0547
Aspartate Transaminase (U/L)	37 (27-57)	36 (27-54)	52.5 (34-87)	<0.001
Alanine Transaminase (U/L)	26 (14-45)	26 (14-45)	24 (15-40)	0.9609
Alkaline Phosphatase (U/L)	72 (60-96)	72 (60-94)	85 (56-163)	0.0770
Lactate Dehydrogenase (U/L)	240.5 (185.5-323.5)	231.5 (181.5-312.5)	311.5 (252-458.5)	<0.001
C-Reactive Protein (mg/L)	16.5 (5.61-56.3)	13 (5.03-41.55)	64.6 (41.9-114)	<0.001
Procalcitonin (ng/mL)	0.08 (0.05-0.19)	0.07 (0.05-0.14)	0.76 (0.02-1.73)	<0.001
Blood Urea Nitrogen (mg/dL)	10.6 (8.4-14.5)	10.2 (8.1-13.2)	17.75 (12.1-32)	<0.001
Creatinine (mg/dL)	0.82 (0.67-1.03)	0.8 (0.66-0.98)	1.21 (0.88-2.12)	<0.001
Na^+ (mmol/L)	136.95 (134.1-139.1)	137.3 (134.6-139.3)	133.85 (132.1-136.9)	<0.001
K^+ (mmol/L)	3.6 (3.4-3.9)	3.6 (3.4-3.9)	3.9 (3.5-4.3)	0.0003
Cl^- (mmol/L)	101 (98-103)	101 (98-103)	97.5 (94-100)	<0.001
$HCO3^-$ (mmol/L)	22.35 (20.2-24)	22.55 (20.6-24.2)	20.25 (18.6-22.2)	<0.001
Anion Gap	14 (12-16)	14 (12-16)	17 (13-21)	0.0005

Hemoglobin A1c (%)	5.9 (5.5-6.7)	5.9 (5.5-6.5)	6.2 (55.5-7.8)	0.231
Lactate (mmol/L)	1.4 (1-1.9)	1.2 (0.9-2)	1.5 (1.2-1.8)	0.339
pH	7.44 (7.40-7.48)	7.45 (7.41-7.47)	7.43 (7.40-7.48)	0.621
pCO2 (mmHg)	29.8 (26.7-33.6)	31.65 (28-33.2)	28.95 (25.8-34.1)	0.296
pO2 (mmHg)	79.95 (61.1-93.9)	77 (56.2-86.1)	81.7 (63.55-112.45)	0.291
SO2 (%)	94.9 (91.8-97.45)	94.65 (90.4-97.05)	95.35 (93.35-97.85)	0.409
**Hematological parameters**				
Prothrombin Time (Secs)	12.6 (11.5-14)	12.7 (11.4-13.7)	12.6 (11.8-15.8)	0.115
Activated Partial Thromboplastin Time (Secs)	25.9 (21.6-29.5)	25.6 (21.6-28.9)	26.6 (21.6-33.9)	0.294
D-dimer (µg/mL)	0.51 (0.3-0.93)	0.46 (0.28-0.83)	1.185 (0.69-3.075)	<0.001
Hb (g/dL)	12.9 (11.6-14.10)	13 (11.8-14.3)	11.45 (8.9-13.7)	0.0007
Hct (%)	38.8 (34.8-42.2)	39.2 (35.5-42.4)	34.4 (26.1-40.2)	0.0003
Red Cell Count (x10^6 cells/ µL)	4.78 (4.26-5.26)	4.8 (4.36-5.29)	4.27 (3.21-5.09)	0.0022
MCV (fL)	82.9 (76.9-86.6)	82.8 (77-86.5)	83.3 (76.1-87.4)	0.726
MCH (pg)	27.8 (25.7-29.4)	27.8 (25.7-29.3)	28.05 (26.1-29.5)	0.566
MCHC (g/dL)	33.4 (32.7-34.1)	33.4 (32.7-34.1)	33.7 (32.8-34.7)	0.181
RDW (%)	13.4 (12.6-14.5)	13.2 (12.6-14.2)	14.6 (13.5-17.2)	<0.001
Platelets (x10^3 cells/ mm^3)	227 (178-285)	231 (183-289)	177.5 (117-232)	<0.001
MPV (fL)	10.2 (9.6-10.9)	10.2 (9.6-10.8)	10.45 (9.9-11.2)	0.047
White Cell Count (x10^3 cells/µL)	6 (4.8-8)	6 (4.7-7.9)	7.15 (5.6-10.8)	0.0100
Neutrophil (%)	65.1 (55-74.7)	63.5 (54.4-72.4)	77.4 (68.5-84.2)	<0.001
Lymphocyte (%)	25.7 (18-35.7)	26.8 (19.5-36.6)	14.55 (9.3-23.3)	<0.001
